# Application of Smoothing Spline in Determining the Unmanned Ground Vehicles Route Based on Ultra-Wideband Distance Measurements

**DOI:** 10.3390/s22218334

**Published:** 2022-10-30

**Authors:** Łukasz Rykała, Andrzej Typiak, Rafał Typiak, Magdalena Rykała

**Affiliations:** 1 Faculty of Mechanical Engineering, Military University of Technology, 00-908 Warsaw, Poland; 2 Faculty of Security, Logistics and Management, Military University of Technology, 00-908 Warsaw, Poland

**Keywords:** UGV, ultra-wideband, UWB, smoothing spline, nonparametric regression, path planning, follow-me, smoothing parameter

## Abstract

Unmanned ground vehicles (UGVs) are technically complex machines to operate in difficult or dangerous environmental conditions. In recent years, there has been an increase in research on so called “following vehicles”. The said concept introduces a guide—an object that sets the route the platform should follow. Afterwards, the role of the UGV is to reproduce the mentioned path. The article is based on the field test results of an outdoor localization subsystem using ultra-wideband technology. It focuses on determining the guide’s route using a smoothing spline for constructing a UGV’s path planning subsystem, which is one of the stages for implementing a “follow-me” system. It has been shown that the use of a smoothing spline, due to the implemented mathematical model, allows for recreating the guide’s path in the event of data decay lasting up to a several seconds. The innovation of this article originates from influencing studies on the smoothing parameter of the estimation errors of the guide’s location.

## 1. Introduction

The term unmanned ground vehicles (UGVs) refers to robots that can travel on land without human operators [1]. In some cases, UGVs can operate autonomously, while in others, operators can control them remotely [2]. In the so called “follow-me” mode, the operator does not have to manually control the platform. This mode allows the vehicle to follow the route set by the guide [3]. Navigating UGVs in “follow-me” mode requires the precise location of the guide to be determined. Guides are responsible for creating paths for UGVs, as mentioned earlier. Maintaining a set distance from the guide and keeping the platform’s heading are the most important aspects of this mode [4]. UGVs should be able to follow the guide in a smooth motion, but if there is an emergency, the guide may stop during movement [5]. It is possible to implement these functionalities using the components of the “follow-me” system, including the guide’s observation subsystem, the path planning subsystem, and the control subsystem [6]. “Follow-me” systems can be divided, inter alia, because of the mode of interaction and degree of autonomy [3]. Mode of interaction refers to the way that the platform interacts with the guide and it can be explicit or implicit. If a human does not directly command the platform, the mentioned mode is explicit. On the other hand, in the case of the degree of autonomy, the most common variant is partial autonomy. Fully autonomous systems use multiple technologies simultaneously and are extremely expensive. The use of “follow-me” systems in dangerous terrain means that the UGV relies heavily on the guide’s movement (implicit mode of interaction and partial autonomy). The platform also does not have to follow the guide in real-time, if it is not necessary in a given situation. To do this, the guide moves first, marking a certain path, then stops and waits for the UGV to reach it. In this case, determining the exact location of the guide is crucial.

Knowing the guide’s location is the basis of the “follow-me” system, and this task is performed within the guide’s observation subsystem [7]. This article is based on the field test results of an outdoor localization subsystem based on ultra-wideband (UWB) technology [8] constituting one of the elements of the “follow-me” system. In order to locate objects using UWB technology, very short data packets are sent wirelessly with a very low power spectral density using the radio energy scattering technique (time of flight). It provides the bandwidth needed to transfer the required amounts of data [9,10].

The mentioned outdoor localization subsystem consists of a total of five UWB modules. Four of them (receivers) were deployed on an existing UGV, while the fifth module (transmitter) was attached to the guide as a part of the developed subsystem. The described subsystem estimates the relative operator’s position based on distance measurements [11,12]. Various technologies are used in commercial “follow-me” system solutions, including UWB [13,14]. Because the main task of the platform in the above-mentioned cases is to keep following the guide (not necessarily along the path indicated by him), a smaller number of receivers is used (usually two), which results in a lower accuracy of the guide’s localization.

Using the guide’s location subsystem, the UGV’s desired route is determined based on the above premise. In the path planning subsystem, successfully calculated guide positions are used to create a route [15,16]. Finally, the planning subsystem aims to provide input signals to the control systems that facilitate the execution of the planned path [17].

The problem of determining the route of a guide’s movement requires solving the problem of fitting a continuous function to a discrete set of the guide’s locations. The numerical methods used in solving the data fitting problem are interpolation and approximation [18]. Interpolation is rarely used in relation to data from experimental measurements because of the presence of disturbances in devices (the interpolation function must pass through the given points) [19]. Approximation, in turn, allows for smoothing and simplifying the course of the analyzed data sets [20]. In addition, this method can be used for large data sets as opposed to interpolation.

The approximation is the problem of describing a data set using approximating functions f(x). When using the approximation methods, a certain set of base functions is assumed, from which the approximating function is defined and the method of its use is determined. The most frequently used form of approximating functions is the so-called general polynomials. The approximation task consists of finding the coefficient values of the generalized polynomial so that the approximating function minimizes the adopted criterion, e.g., the sum of the squared differences. The concept of approximation is also closely related to the concept of regression, which is a solution to the problem of point approximation for a data set, but its final result is, apart from the sought function coefficients, also a function model. Regression allows for determining the symbolic form of the function, which, meeting the adopted criteria, reflects the individual values of the dependent variable for the previously defined set of independent variable values [18,21].

Regression can be divided into two main types: parametric and non-parametric [22]. Some sources also distinguish semiparametric methods, which are rarely used [22]. Mentioned regression models are chosen according to the prior knowledge of the functional form and the random error distribution. The most important criterion, however, is the knowledge of the functional form. If it is known, parametric regression will be able to fit the data. The parametric approach requires knowledge of a mathematical model, which can be simple (e.g., linear regression), and its parameters are assumed directly [23]. Because the guide can move in any direction, marking the platform’s path, it is impossible to make any assumptions about its route. Therefore, the application of the parametric regression approach in the analyzed case becomes very difficult to implement.

There are also non-parametric regression methods in which the form of the model is not clearly defined and their parameters are not taken directly [24,25]. Nonparametric regression methods, including Kernel regression [26,27], LOWESS (locally weighted scatterplot smoothing) [22], and smoothing spline [28,29,30,31], have an extensive form of a mathematical model. Nonparametric regression models are much more flexible and computationally complex compared with parametric models. In addition, they avoid erroneous fitting results when the wrong model is used. The result of the application of the above-mentioned methods is not a mathematical relationship; therefore, the mentioned results are also difficult to export [22].

To determine the guide’s route, the desired method should be characterized by a moderate computational complexity, have the ability to parametrically shape the smoothing of the coordinates of the guide’s location, and be able to estimate the missing coordinates based on the present values.

Kernel regression smoothing is a technique that uses kernel functions as a weighing function for developing a non-parametric regression model. It can be applied to high-dimensional data sets and it can be used for fitting the data without making any distributional assumptions about it. It is more flexible than other non-parametric approaches, but it does not have any direct smoothing parameter [32,33]. In turn, the LOWESS method is based on the simplicity of linear least squares regression, which makes it highly exposed to the effects of outliers in the data set [34]. Similar to the mentioned Kernel regression method, LOWESS does not have a direct smoothing parameter. Additionally, the Kernel method is much more computationally complex than the LOWESS method.

However, the only analyzed non-parametric method that meets the mentioned criteria is a smoothing spline. Among other methods of non-parametric regression, it is distinguished by a lower computational complexity and the presence of a direct parameter smoothing the given waveform. Moreover, it is not exposed to the effects of outliers in the data set. Therefore, a smoothing spline was chosen as the method for calculating the guide’s route.

Researchers have focussed on the study of path planning algorithms of autonomous robots (which can also work under the “follow-me” system) using various modern methods, including smoothing splines [35]. No studies were found on the use of the smoothing spline method in the context of planning the movement of the UGV (or robot in general) as part of the “follow-me” system, hence the article is an innovation in the field. Moreover, no studies were found on the influence of the smoothing parameter on the estimation on the guide’s path.

The aim of the article is to determine the route of the guide using a smoothing spline based on the designated locations using UWB technology. In order to generate a smoothing spline, it is necessary to specify a value for the smoothing parameter. Because of this, it is necessary to conduct research on the influence of the aforementioned parameter on the estimation of the guide’s route and select a value that meets the selected evaluation criterion, e.g., minimization of the sum of errors.

## 2. Materials and Methods

A smoothing spline (so-called polynomial spline or polynomial smoothing curve) is a k-th degree piecewise polynomial that has k−1 continuous derivatives. The mentioned curve is most often used to approximate a data set of points with cubic polynomials (3rd order, two continuous derivatives). The advantage of using the mentioned curve is the possibility of reaching a compromise between two opposing aims:

- fitting the value of the dependent variable to the set of independent variable values,
- smoothing the course of the value of the dependent variable (minimizing the curvature of the trajectory and its acceleration) [22].

In order to describe the mathematical model of a polynomial curve, the first step is to define the vectors of the dependent variables q and the independent variables t:

(1) $$q={\left[q_{0},q_{1},q_{2},\ldots ,q_{n}\right]}^{T}$$

(2) $$t={\left[t_{0},t_{1},t_{2},\ldots ,t_{n}\right]}^{T}$$

Then, the parameters of the aforementioned curve s(t) are obtained by minimizing the dependence S:

(3) $$S=\lambda \overset{n}{\underset{k=1}{\sum }}{\left[s\left(t_{k}\right)-q_{k}\left(t_{k}\right)\right]}^{2}+\left(1-\lambda \right)\int_{t_{0}}^{t_{n}}\ddot{s}{\left(t\right)}^{2}\mathrm{dt}$$

where λ∈[0,1]—the so-called smoothing parameter, $s_{i}\left(t_{i}\right)={\left[s_{1}\left(t_{1}\right),\ldots ,s_{n}\left(t_{n}\right)\right]}^{T}$—smoothing spline function parameters [22].

The curve parameters are determined for each node, while the nature of its estimation is determined by the smoothing parameter λ, which takes values in the range of [0, 1]. In extreme cases, when λ = 0, a linear approximation is obtained using the least squares method, and for λ = 1, interpolation using a cubic polynomial is obtained. Thus, when λ tends to zero, the smoothing effect of the course is maximized, while when λ tends to 1, the fidelity of the mapping of the set of points is maximized [36].

### 2.1. Determination of the Value of the Smoothing Parameter

The present article is a direct extension of the research carried out in [8]. Moreover, the results of the mentioned research form the basis of the article.

A Decawave TREK1000 evaluation kit [37], which consists of five UWB modules, was used in the research. The developed system consists of five modules: four receivers called anchors and a transmitter called a tag, carrying out continuous distance measurements with a frequency of 10 Hz. UWB modules were placed on the UGV (anchors) and the guide (tag). The accuracy of a single anchor–tag measurement is approximately 10 cm using the two-way ranging time-of-flight (TOF) technique. The UWB system provides information about the distance from the individual anchors to the tag.

**Figure 1:**
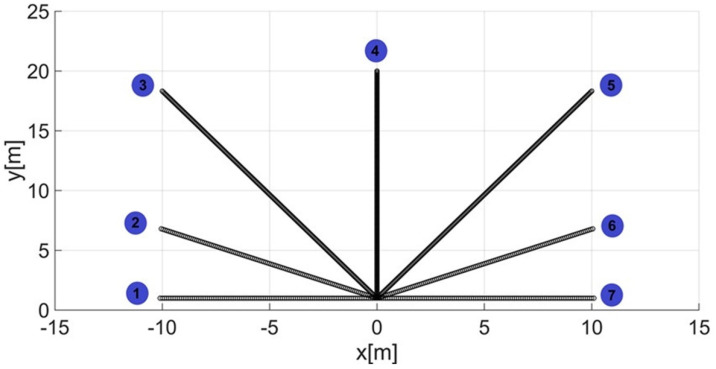
Guide paths 1−7 with the adopted xy coordinate system [6].

For the research, it was assumed that the human guide moves along seven rectilinear paths inclined at an angle of 0°, 30°, 60°, 90°, 120°, 150°, and 180°, respectively, to the *x*-axis of the xy coordinate system in the area satisfying the following inequalities: −10 m < x < 10 m and 0 m < y < 20 m (Figure 1).

**Figure 2:**
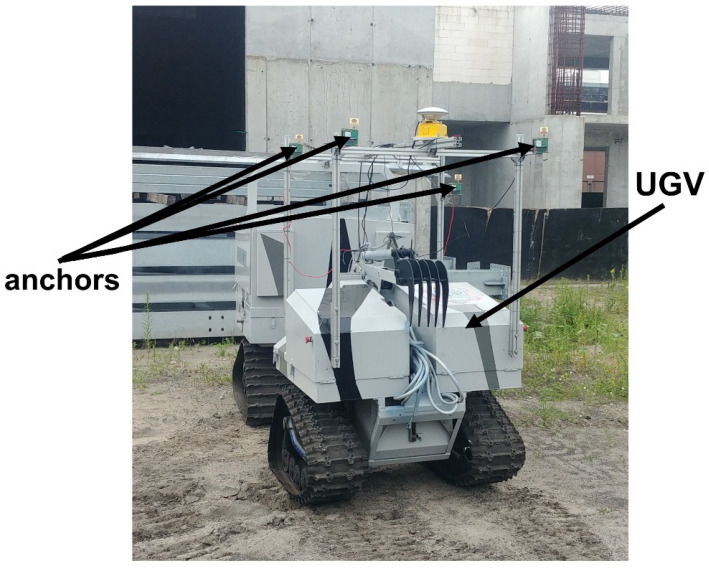
Configuration of UWB modules on a UGV.

Next, guide paths no. 1–7 (Figure 1) were recreated with the assumption that the UGV remains stationary. Moreover, the guide was supposed to turn 180 around its axis after reaching the turning point and then return along the same track to the starting point. The arrangement of the anchors on the UGV is shown in Figure 2 (spatial configuration of the anchors for the correct operation of the location subsystem).

**Figure 3:**
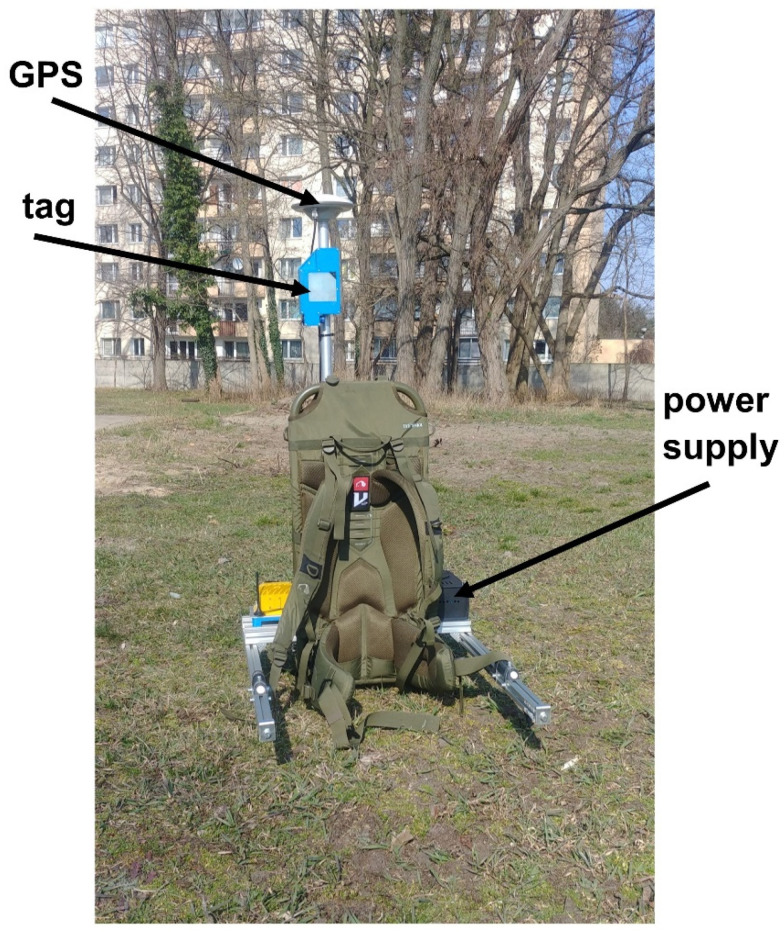
Mobile UWB guide location kit.

During the movement, the guide carried a specially made frame with the necessary equipment (mobile location kit): a UWB tag, a GPS module, and a power supply system (Figure 3).

In order to determine the location errors of the results obtained with the UWB technology, SwiftNav DURO satellite receivers operating in the RTK mode were used (error: 1 cm horizontally and 1.5 cm vertically) [38].

**Figure 4:**
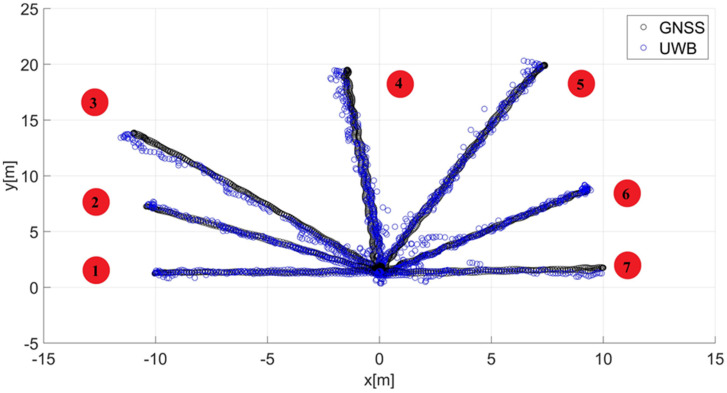
The results of the guide’s location for routes no. 1−7 using UWB technology along with the reference positions obtained using the GNSS module. Own elaboration based on [8].

The starting point of the article is the final results of research on the described location system based on UWB technology using the nonlinear programming (NLP) method based on the Levenberg–Marquardt (LM) algorithm [8]. The mentioned results (Figure 4) are the basis for the further determination of the guide’s path.

In order to calculate the guide’s route, first, the smoothing parameter value should be specified. Then, after determining said parameter, it becomes possible to implement a polynomial curve for the results of the experimental research of the location subsystem.

The following values were adopted to evaluate the obtained results:

- total error

(4) $$e_{c}\left(t\right)=\sqrt{e_{x}{\left(t\right)}^{2}+e_{y}{\left(t\right)}^{2}}$$

where $e_{x}\left(t\right)\;$ is the error of mapping the guide’s location on the *x*-axis of the coordinate system at time t, $e_{y}\left(t\right)$ is the error of mapping the guide’s location on the *y*-axis of the coordinate system at time t.

- quality indicator

(5) $$Q=\sum^{\;}e_{c}\left(t\right)$$

- mean value of the quality indicator

(6) $$Q_{\mathrm{av}}=\frac{\sum^{\;}e_{c}\left(t\right)}{l}$$

where l is the number of distance measurements [6].

Most often, the value of the smoothing parameter is determined using the following relationship:

(7) $$\lambda_{p}=\frac{1}{1+\frac{p^{3}}{6}}$$

where p is the average spacing of data points [36].

The smoothing parameter calculated according to dependence 7 (p = 0.1) is approximately λ_p_ = 0.99. Such a high value indicates the maximization of data fidelity, which, due to the presence of disturbances resulting in localization errors, is not always the most recommended solution. Therefore, the influence of the smoothing parameter on the guide’s route estimation is determined in the article. In order to achieve the above-mentioned purpose and to select the final value of the smoothing parameter smoothing splines were calculated for the smoothing parameters λ ∈ [0.05; 0.1; 0.15,…, 0.95] and routes no. 1–7 (Figure 4). Then, for each value of the smoothing parameter, the quality indicator Q and finally the average value of quality indicator Q_av_ were determined. Additionally, for each case, the mean square value of the acceleration a_RMS_ (the second derivative of the dependent variable) was also determined. Based on the minimization of the average quality indicator, the final value of the smoothing parameter was selected. The knowledge of a chosen smoothing parameter made it possible to determine the estimation of guide routes no. 1–7 using a smoothing spline. Matlab/Simulink software with the Curve Fitting Toolbox was used in the research.

### 2.2. The Influence of the Smoothing Parameter on the Guide’s Path Estimation

**Figure 5:**
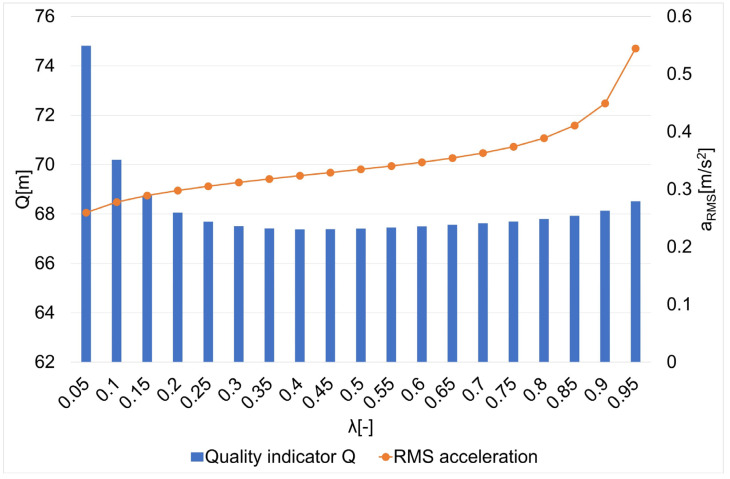
The values of the quality indicator Q and the mean square value of acceleration a_RMS_ for the smoothing parameters in the case of path no. 1 [6].

**Figure 6:**
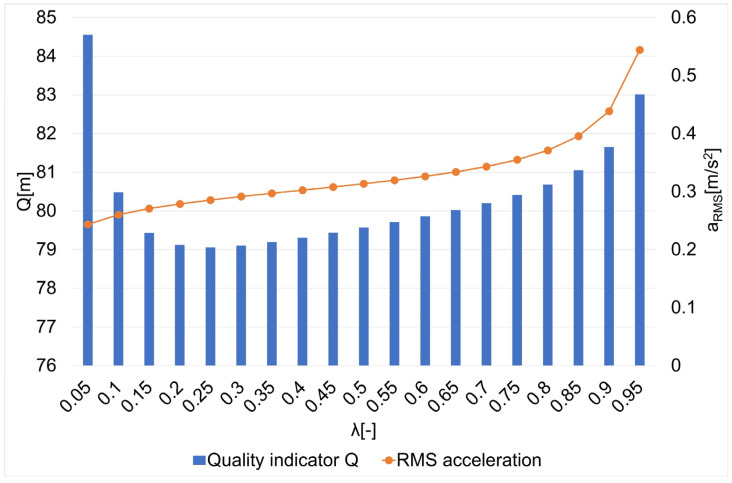
The values of the quality indicator Q and the mean square value of acceleration a_RMS_ for the smoothing parameters in the case of path no. 2 [6].

**Figure 7:**
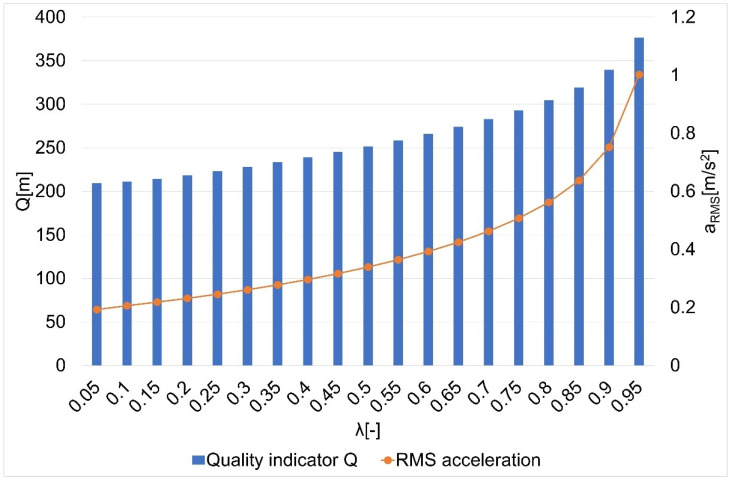
The values of the quality indicator Q and the mean square value of acceleration a_RMS_ for the smoothing parameters in the case of path no. 3 [6].

**Figure 8:**
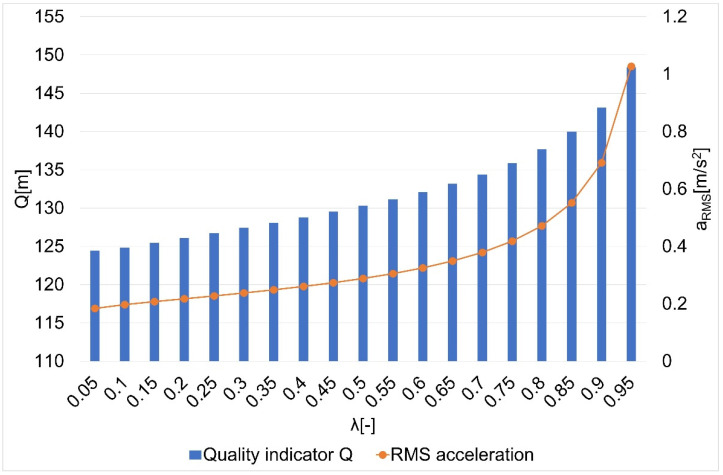
The values of the quality indicator Q and the mean square value of acceleration a_RMS_ for the smoothing parameters in the case of path no. 4 [6].

**Figure 9:**
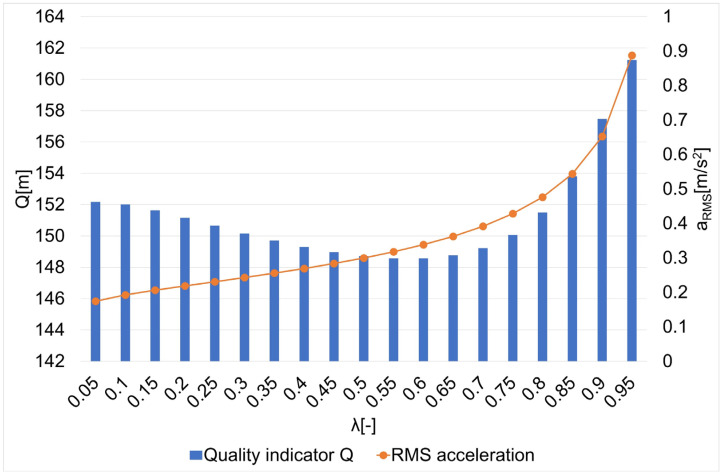
The values of the quality indicator Q and the mean square value of acceleration a_RMS_ for the smoothing parameters in the case of path no. 5 [6].

**Figure 10:**
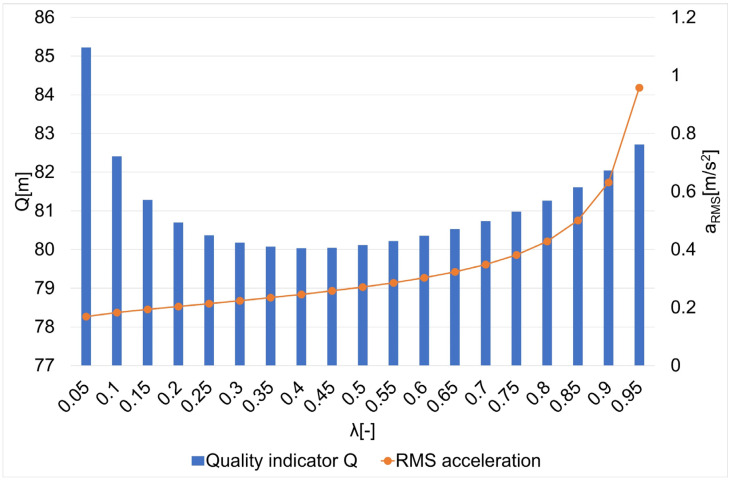
The values of the quality indicator Q and the mean square value of acceleration a_RMS_ for the smoothing parameters in the case of path no. 6 [6].

**Figure 11:**
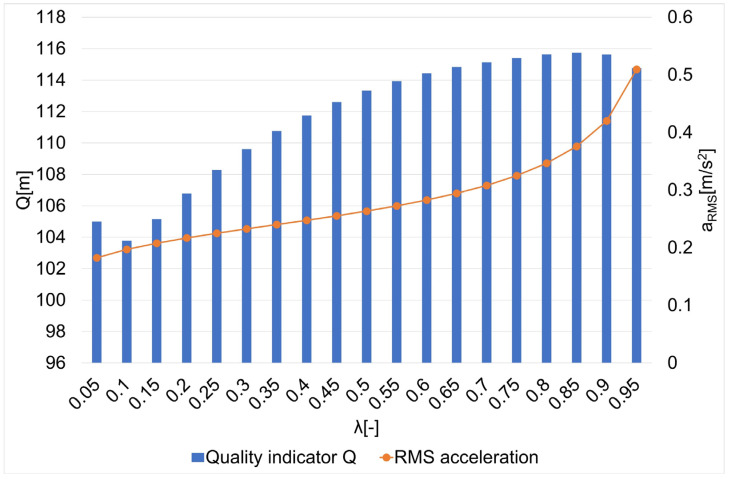
The values of the quality indicator Q and the mean square value of acceleration a_RMS_ for the smoothing parameters in the case of path no. 7 [6].

**Figure 12:**
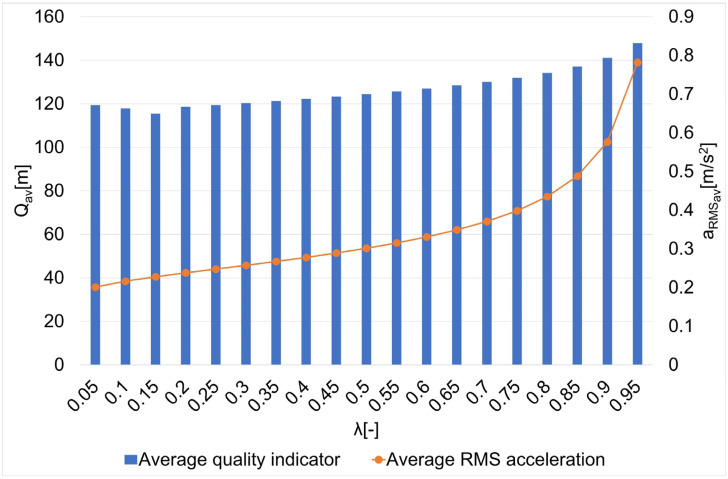
The values of the average quality indicator Q and the average mean square value of acceleration a_RMS_ for the smoothing parameters in the case of paths no. 1–7 [6].

The results of the research on the influence of the smoothing parameter on the values of the Q quality indicator and the average square acceleration values for guide routes no. 1–7 are shown in Figure 5, Figure 6, Figure 7, Figure 8, Figure 9, Figure 10 and Figure 11. Figure 12 shows the values of the average quality indicator and the mean square root acceleration values for all of the considered results.

The values of certain quality indicators Q (Figure 5, Figure 6, Figure 7, Figure 8, Figure 9, Figure 10 and Figure 11) obtain values ranging from about 79 m (traffic path no. 2) to about 375 m (traffic path no. 3). Determination of the average value of the quality indicator Q_av_ allows for generalizing the obtained results and for determining the final value of the smoothing parameter minimizing the indicator, which is the sum of the total errors (dependence 5). On the other hand, the courses of the mean square values of acceleration a_RMS_ for all of the considered paths (Figure 5, Figure 6, Figure 7, Figure 8, Figure 9, Figure 10 and Figure 11) show an increasing trend obtaining the minimum quality indicator Q for the value of λ = 0.05 and the maximum for the value of λ = 0.95.

The Q_av_ indicator obtained the minimum value for the smoothing parameter λ = 0.15 (Figure 12), which was adopted in the further part of the research as the smoothing parameter of the smoothing spline.

## 3. Results

**Figure 13:**
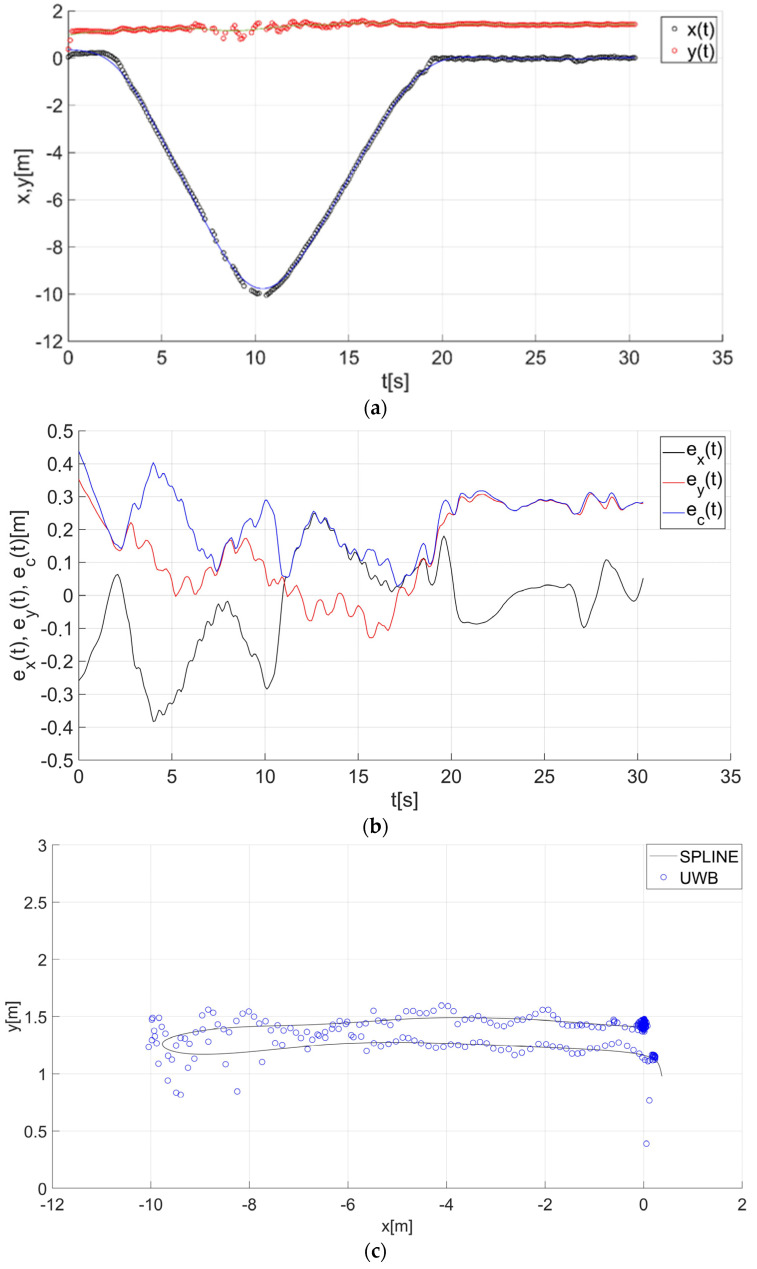
Results for the estimation of guide path no. 1 with the use of the smoothing spline: (**a**) the course of the guide’s location coordinates x(t), y(t) with their continuous estimates, (**b**) the course of the estimated location errors e_x_(t), e_y_(t), e_c_(t), (**c**) guide’s location along with the path estimation [6].

**Figure 14:**
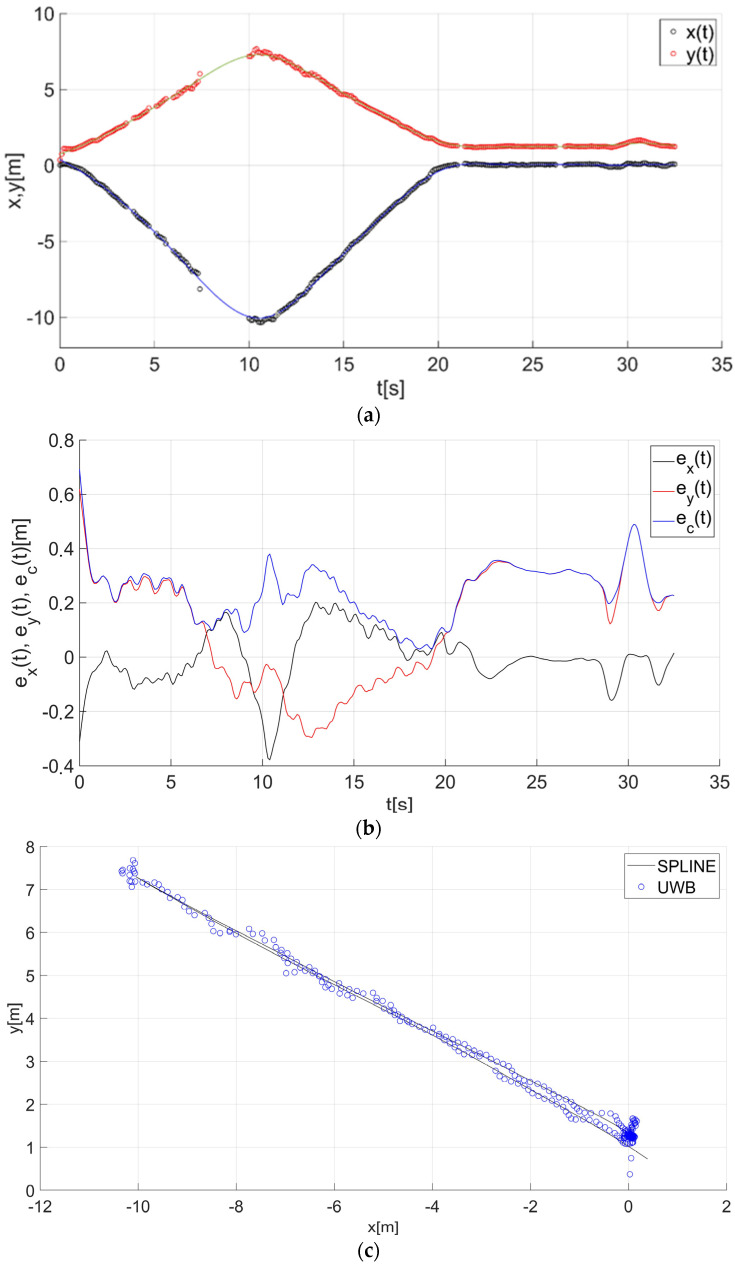
Results for the estimation of guide path no. 2 with the use of the smoothing spline: (**a**) the course of the guide’s location coordinates x(t), y(t) with their continuous estimates, (**b**) the course of the estimated location errors e_x_(t), e_y_(t), e_c_(t), (**c**) guide’s location along with the path estimation [6].

**Figure 15:**
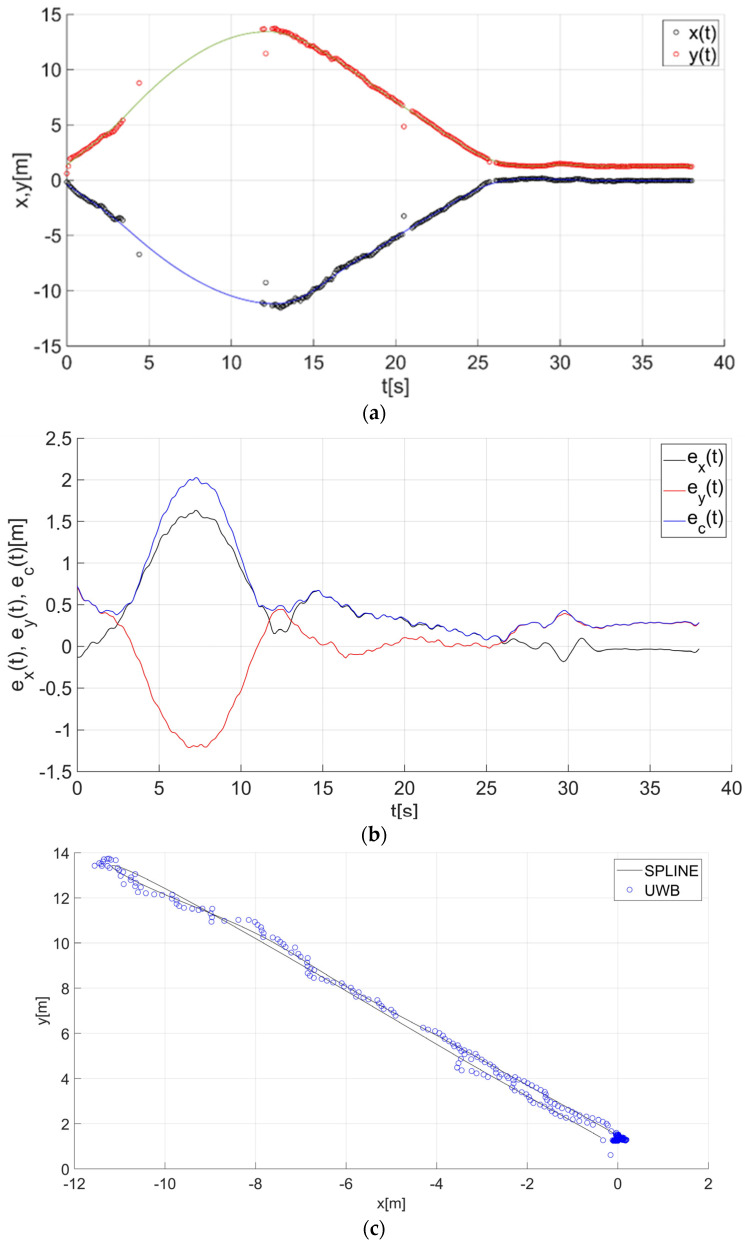
Results for the estimation of guide path no. 3 with the use of the smoothing spline: (**a**) the course of the guide’s location coordinates x(t), y(t) with their continuous estimates, (**b**) the course of the estimated location errors e_x_(t), e_y_(t), e_c_(t), (**c**) guide’s location along with the path estimation [6].

**Figure 16:**
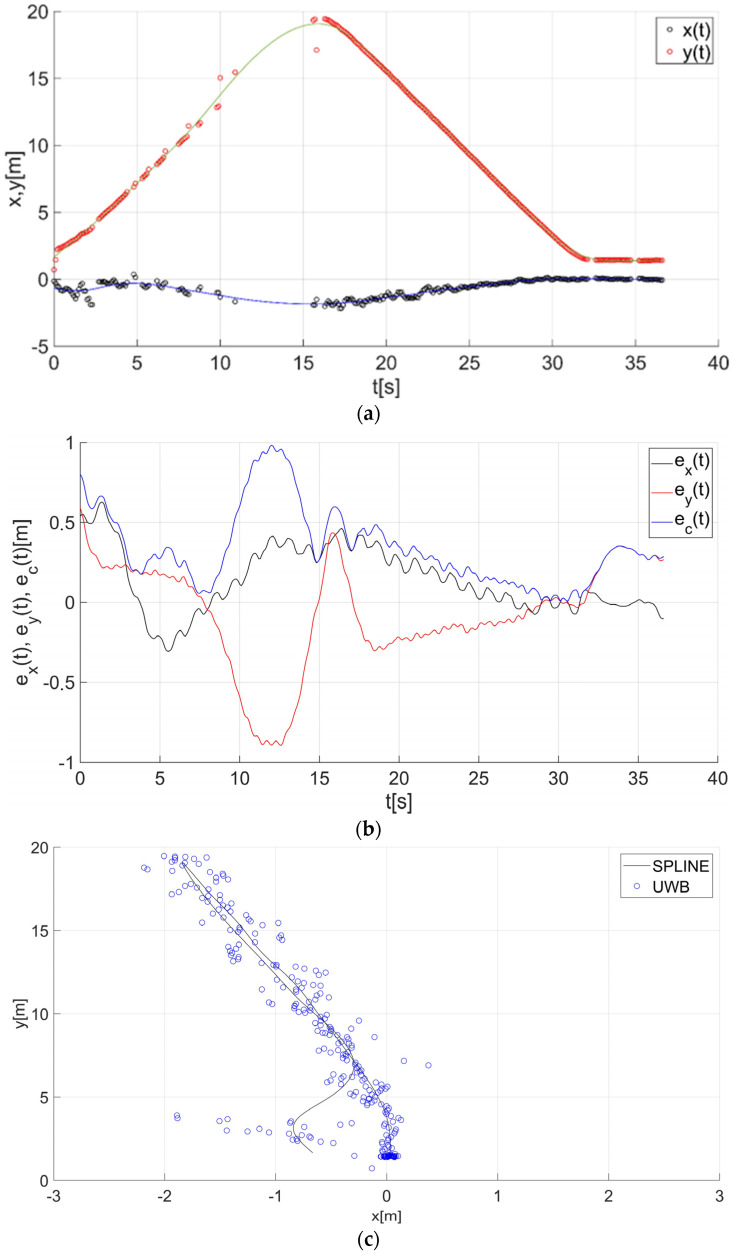
Results for the estimation of guide path no. 4 with the use of the smoothing spline: (**a**) the course of the guide’s location coordinates x(t), y(t) with their continuous estimates, (**b**) the course of the estimated location errors e_x_(t), e_y_(t), e_c_(t), (**c**) guide’s location along with the path estimation [6].

**Figure 17:**
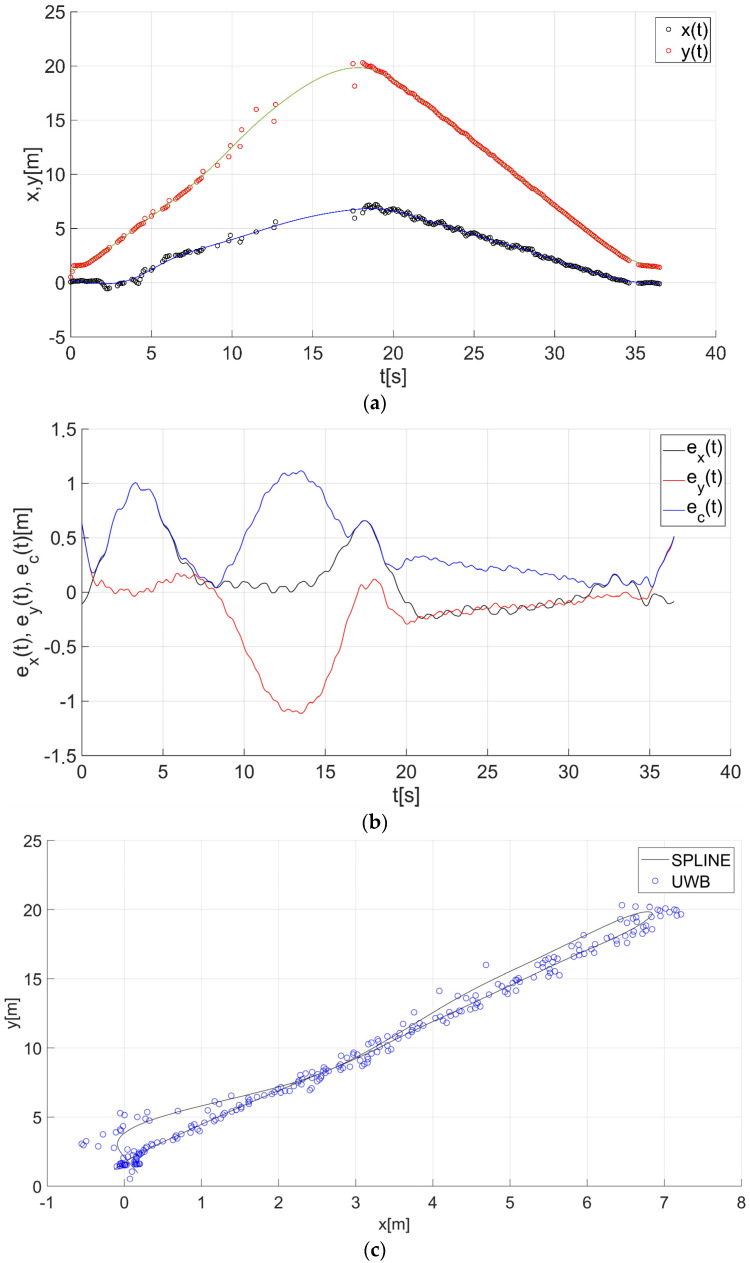
Results for the estimation of guide path no. 5 with the use of the smoothing spline: (**a**) the course of the guide’s location coordinates x(t), y(t) with their continuous estimates, (**b**) the course of the estimated location errors e_x_(t), e_y_(t), e_c_(t), (**c**) guide’s location along with the path estimation [6].

**Figure 18:**
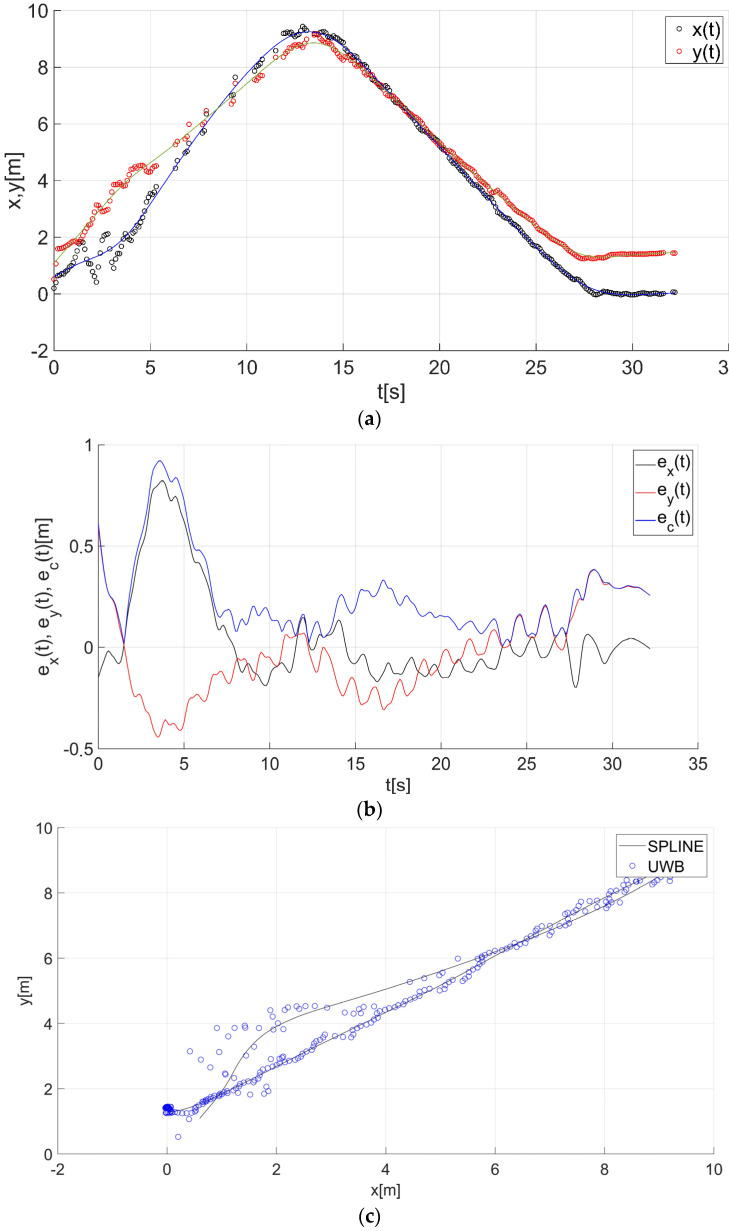
Results for the estimation of guide path no. 6 with the use of smoothing spline: (**a**) the course of the guide’s location coordinates x(t), y(t) with their continuous estimates, (**b**) the course of the estimated location errors e_x_(t), e_y_(t), e_c_(t), (**c**) guide’s location along with the path estimation [6].

**Figure 19:**
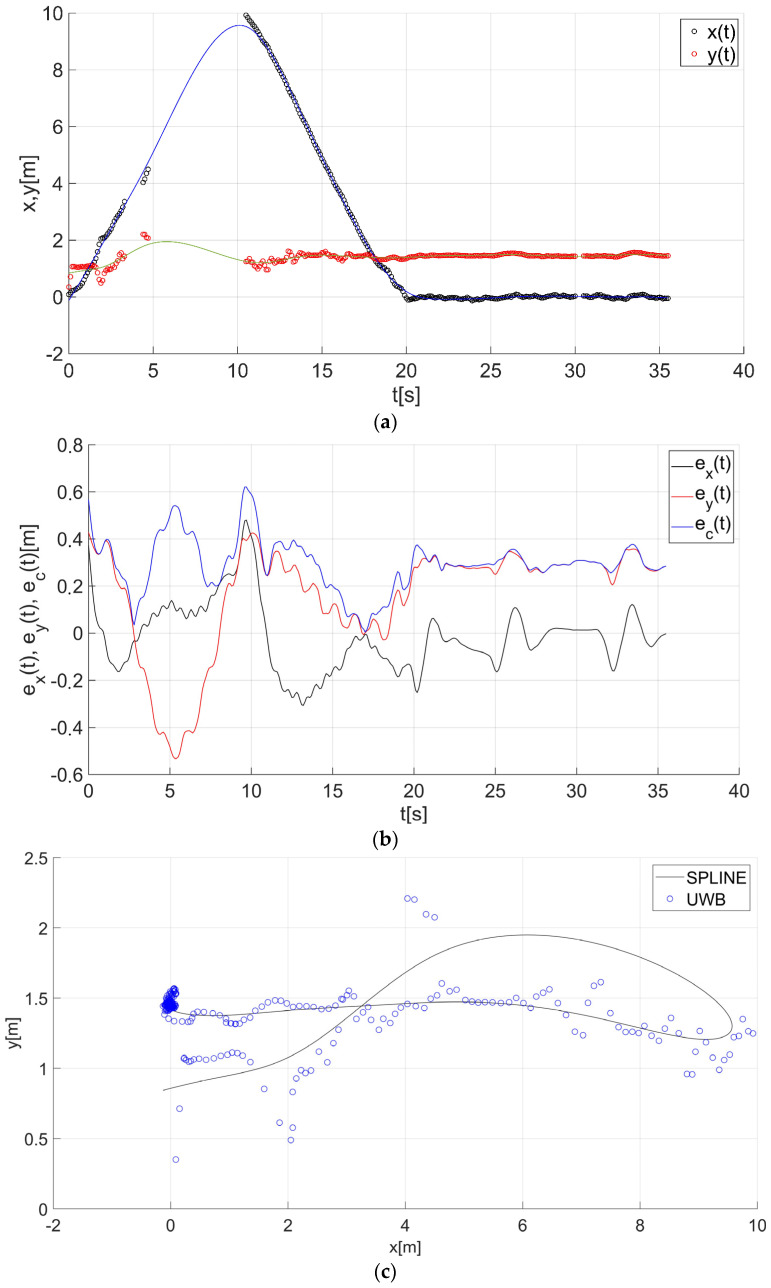
Results for the estimation of guide’s path no. 7 with the use of smoothing spline: (**a**) the course of the guide’s location coordinates x(t), y(t) with their continuous estimates, (**b**) the course of the estimated location errors e_x_(t), e_y_(t), e_c_(t), (**c**) guide’s location along with the path estimation [6].

After determining the value of the smoothing parameter λ (λ = 0.15), the guide’s route for paths 1–7 (Figure 1) was determined using a smoothing spline. Figure 13a, Figure 14a, Figure 15a, Figure 16a, Figure 17a, Figure 18a, and Figure 19a show the x(t) and y(t) coordinates of the guide’s location along with their estimates for the considered cases. Errors in determining the guide’s route using the mentioned method are shown graphically in Figure 13b, Figure 14b, Figure 15b, Figure 16b, Figure 17b, Figure 18b, and Figure 19b. Moreover, Figure 13c, Figure 14c, Figure 15c, Figure 16c, Figure 17c, Figure 18c, and Figure 19c show the guide’s location along with the estimation of its route with the use of a smoothing spline concerning all of the analyzed paths.

**Figure 20:**
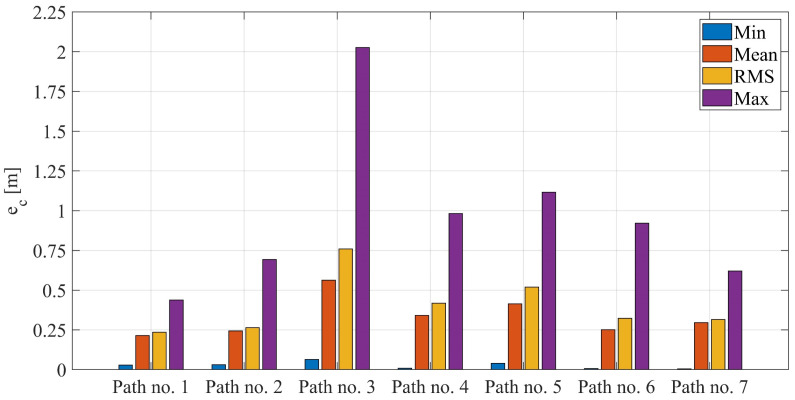
Basic descriptive statistics: minimum, mean, root mean square, and maximum of total errors for paths no. 1–7.

The courses of the estimated coordinates of the guide’s position at x(t), y(t) are presented as a function dependent of time in Figure 13a, Figure 14a, Figure 15a, Figure 16a, Figure 17a, Figure 18a, and Figure 19a, while in Figure 13c, Figure 14c, Figure 15c, Figure 16c, Figure 17c, Figure 18c, and Figure 19c they are presented as a function independent of time in the form of y(t) = f(x(t)). In turn, location errors on the *x* and *y* axes of the xy coordinate system and the total errors are shown in Figure 13b, Figure 14b, Figure 15b, Figure 16b, Figure 17b, Figure 18b, and Figure 19b. In all of the cases, the decay of signals can be noticed (Figure 13a, Figure 14a, Figure 15a, Figure 16a, Figure 17a, Figure 18a, and Figure 19a), which increases the total errors of the estimated path. Basic descriptive statistics of the total errors for all of the considered paths are shown in Figure 20.

The error values do not exceed the following values: minimum 0.07 m, mean 0.57 m, RMS 0.76 m, and maximum 2.03 m (Figure 20). The largest decay of location signals is noticeable in the case of path no. 3 (decay lasting approx. 10 s, Figure 15a), and it translates into the above-mentioned maximum values of the total errors (Figure 15b and Figure 20). However, even in this case, the estimated trajectory retains the shape of the reference trajectory (Figure 15c).

## 4. Conclusions

The method of determining the guide’s route using a smoothing spline was discussed in the article. In the context of the “follow-me” systems, there has been no systematic study of the application of the smoothing spline method for planning the movement of the UGV, so the article represents an innovation in the field.

The influence of the smoothing parameter on the estimation of the guide’s route was also determined in the article. In addition, no studies were found that examined the impact of the described parameter on path estimation.

As a result of the implemented mathematical model, it has been shown that the smoothing spline can recreate the path of the guide after a 10 s period of decay of the guide’s localization results. The occurrence of the aforementioned guide’s location decays increased the total errors for estimating the guide’s route.

The value of the smoothing parameter affects the guide’s route estimation. The choice of the final value of the smoothing parameter requires additional experimental studies. The dependence existing in the literature [36] that allows for automatically determining the value of the smoothing parameter for any data is not universal and it is only a preliminary estimate of the parameter value. It always has to be adapted to the application under consideration.

The value of the smoothing parameter also affects the estimation of the linear acceleration of the guide. An increase in the value of the smoothing parameter increases the mean square value of the linear acceleration of the guide.

## Data Availability

The data presented in this study are available on request from the corresponding author.
